# Biological, Environmental, and Social Determinants of Buruli Ulcer in Africa: Their Interplay Across Disease Severity and Patient Outcome

**DOI:** 10.1002/puh2.70389

**Published:** 2026-10-04

**Authors:** Gudisa Bereda

**Affiliations:** ^1^ Department of Pharmacy Marie Stopes International Ethiopia (MSIE) Ambo Ethiopia

**Keywords:** biological determinants, Buruli ulcer, environmental determinants, health outcomes, *Mycobacterium ulcerans*, social determinants

## Abstract

Buruli ulcer (BU), caused by *Mycobacterium ulcerans*, is a neglected tropical disease influenced by interacting biological, environmental, and social determinants in Africa. Biological factors include mycolactone‐mediated immunosuppression, host immune response, lesion severity, age, HIV infection, genetic susceptibility, and delayed diagnosis. BU primarily affects children under 15 years, whereas HIV co‐infection may contribute to more severe outcomes. Early rifampicin‐based antibiotic therapy improves healing and reduces complications, whereas delayed treatment is associated with larger lesions, increased need for surgery, and long‐term disability. Environmental factors, including aquatic exposure, rainfall, flooding, land‐use changes, and ecological disturbances, may influence the distribution and exposure patterns of *M. ulcerans*. However, specific pathways of human transmission remain uncertain. Social determinants such as poverty, stigma, low education, gender roles, spiritual beliefs, reliance on traditional healers, and limited healthcare access contribute to delayed care‐seeking. In Ghana, the median time to treatment was 25 days for pre‐ulcer lesions and 204 days for ulcerative disease. BU occurs in at least 32 countries, with prevalence ranging from 3.2 to 26.9 per 10,000 population in endemic areas. Integrated strategies combining early detection, antibiotic therapy, wound care, physiotherapy, psychosocial support, stigma reduction, and community engagement are essential.

## Graphical abstract

Buruli ulcer is caused by *Mycobacterium ulcerans* and its toxin, mycolactone. Disease outcomes are influenced by biological, environmental, and social determinants. Biological factors include immune response and lesion severity. Environmental factors include aquatic exposure, rainfall, flooding, and ecological changes. Social factors include poverty, stigma, low education, and limited healthcare access. These factors can delay care‐seeking and diagnosis. Delayed treatment can lead to larger lesions, poor healing, surgery, and disability. Early detection, antibiotics, wound care, physiotherapy, psychosocial support, and community engagement can improve healing and reduce complications and disability.

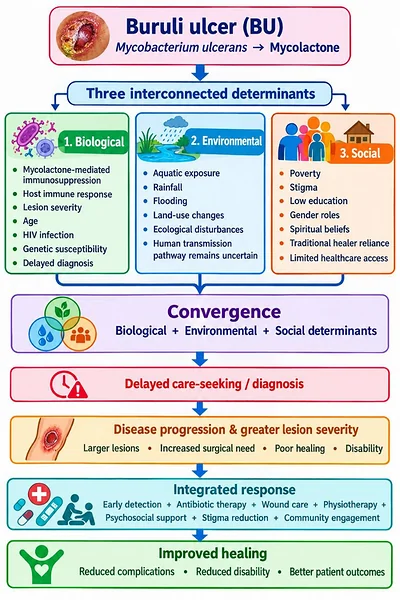

## 1 Introduction

Buruli ulcer (BU) is a chronic, necrotizing skin and soft tissue infection caused by *Mycobacterium ulcerans* and classified by the World Health Organization (WHO) as a neglected tropical disease (NTD) [1]. The disease is most prevalent in rural and resource‐limited regions of West and Central Africa, where environmental exposure to slow‐moving water bodies and poor socioeconomic conditions contribute to transmission [2]. Although the global prevalence of BU is lower than other NTDs, it remains highly endemic in specific African countries, disproportionately affecting children and economically disadvantaged populations [3]. In endemic communities, delayed diagnosis is common, and advanced lesions (Categories II and III) are frequently reported, increasing disease severity, risk of disability, and long‐term complications [4]. Clinically, BU typically presents as painless nodules, plaques, or edema that may progress to extensive ulceration due to the cytotoxic effects of mycolactone, a toxin produced by *M. ulcerans* [5].

Severe cases can result in permanent scarring, contractures, bone involvement, and functional impairment [6]. Although antibiotic therapy has significantly improved clinical cure rates, the severity of lesions at presentation often determines long‐term physical outcomes [7]. However, clinical indicators alone do not fully capture the overall burden of disease. Beyond biological mechanisms, social determinants play a critical role in shaping disease prevalence, severity, and recovery outcomes [8]. Poverty, limited access to healthcare facilities, low health literacy, gender inequality, and reliance on traditional healing practices contribute to delayed health‐seeking behavior, thereby increasing lesion size and complication rates [9]. Stigma and misconceptions surrounding BU frequently lead to social isolation, educational disruption, income loss, and psychological distress [10].

In several endemic settings, the prevalence of depression, anxiety, and reduced health‐related quality of life (HRQoL) among affected individuals is substantial, particularly in cases with severe or visible deformities [11]. Environmental factors may contribute to geographic variation in BU by affecting the abundance of *M. ulcerans* and opportunities for human contact with potentially contaminated environments [12]. Changes in water systems, precipitation, flooding, temperature, vegetation, land use, and ecosystem disturbance may alter conditions associated with disease occurrence [13]. Nevertheless, these relationships are inconsistent across endemic regions, and the mechanisms through which humans become exposed to *M. ulcerans* remain incompletely defined. Biological and social determinants of BU are deeply interconnected [9]. Socioeconomic disadvantage can increase exposure risk and delay healthcare‐seeking, worsening lesion severity and clinical outcomes [14]. Environmental exposures may also interact with biological susceptibility and social conditions by influencing patterns of human exposure [15].

In turn, severe disease may reinforce stigma, limit social participation, and perpetuate economic hardship, creating a cycle of vulnerability [16]. Children and women are particularly affected due to structural inequalities and caregiving burdens [17]. Factors such as host immune response, lesion severity, treatment timing, poverty, health literacy, cultural beliefs, environmental exposure, and access to care interact to influence both biological and psychosocial outcomes [18]. Early detection and supportive community environments can mitigate clinical complications and psychosocial distress, highlighting the need for comprehensive, patient‐centered interventions [9]. Despite growing awareness, existing research often treats biological severity and social determinants as separate domains.

Environmental determinants are also insufficiently integrated into existing frameworks. There remains a critical need for integrative approaches that examine how pathophysiology, lesion severity, treatment timing, stigma, poverty, healthcare access, and environmental exposure influence health outcomes in BU [19]. Such integration is particularly relevant in African contexts, where structural inequities and health system constraints strongly shape patient experiences [9]. Considering these determinants together is important because biological disease processes do not occur independently of the social and environmental conditions in which patients live and receive care [12]. Environmental conditions may shape exposure opportunities and disease distribution, although their specific contribution to human transmission remains uncertain [20]. An integrated perspective can therefore provide a more complete understanding of BU outcomes than considering these determinants separately [12].

This review integrates biological, environmental, and social determinants of BU in Africa, demonstrating how infection severity, environmental exposure, delayed treatment, and social stigma influence health outcomes. It examines pathogen and host factors alongside environmental influences and psychosocial effects, including anxiety, depression, and reduced HRQoL. The synthesis underscores the importance of multidisciplinary interventions, such as community rehabilitation, psychosocial support, environmentally informed prevention, and early detection programs.

## 2 Materials and Methods

### 2.1 Study Design

This study is a narrative review design used to examine the biological, environmental, and social determinants influencing BU outcomes in African populations. Evidence from quantitative, qualitative, observational, clinical, and community‐based studies was synthesized to provide a broad understanding of lesion severity, functional impairment, psychosocial burden, and HRQoL.

### 2.2 Search Strategy

A literature search was conducted using PubMed, Scopus, Web of Science, and Google Scholar. Additional studies were identified through manual searches of reference lists from eligible articles and related reviews. The search was designed to identify literature addressing the biological determinants, environmental determinants and transmission ecology, social determinants, clinical manifestations, functional consequences, psychosocial impacts, and HRQoL outcomes associated with BU in African populations. Search terms included “Buruli ulcer,” “*Mycobacterium ulcerans*,” “biological determinants,” “environmental determinants,” “social determinants,” “stigma,” “quality of life,” “health‐related quality of life,” “lesion severity,” “functional impairment,” “immune response,” and “health outcomes.” These terms were combined using Boolean operators (“AND”/“OR”) and adapted as necessary to the search requirements of each database. The search covered literature published from January 2000 to April 2026 to capture both established and more recent evidence.

### 2.3 Eligibility Criteria

Studies were included if they focused on BU in African populations and involved children, adolescents, adults, or older adults. Eligible studies investigated biological determinants such as lesion size, bacterial burden, mycolactone activity, disease stage, inflammatory markers, or host immune response. Studies examining environmental determinants, including aquatic habitats, water exposure, climate variability, land‐use change, ecological disturbance, wildlife or insect associations, and other environmental factors potentially related to BU occurrence or transmission, were also included. Studies examining social determinants, including stigma, gender roles, socioeconomic status, education, healthcare access, treatment delay, social support, and cultural beliefs, were also included. Studies were required to report outcomes related to lesion severity, disability, functional impairment, psychosocial well‐being, treatment outcomes, or HRQoL. Quantitative, qualitative, mixed‐methods, observational, and community‐based studies were eligible. Studies conducted outside Africa or lacking relevant outcomes were excluded. Editorials, commentaries, conference abstracts without sufficient data, and inaccessible full texts were also excluded.

### 2.4 Study Selection

Titles and abstracts identified through database searches were screened for relevance. Full‐text articles of potentially eligible studies were then assessed using the predefined inclusion and exclusion criteria. Duplicate records were removed before final selection. Only studies meeting all eligibility criteria were included in the narrative synthesis. Reference‐list searches were assessed using the same criteria. The selection process aimed to ensure representation from different African regions and age groups.

### 2.5 Data Extraction

Data were extracted using a standardized framework developed for this review. Extracted information included author names, publication year, country or region, study design, study population, determinants investigated, outcomes assessed, and key findings related to BU severity, disability, HRQoL, and treatment outcomes. Information on age‐specific and lifespan‐related impacts was also collected to identify developmental differences in disease burden and psychosocial effects.

### 2.6 Data Synthesis and Analysis

Included studies were grouped according to biological, environmental, and social determinants. Because of variations in study design, population characteristics, and outcome measures, meta‐analysis was not feasible. Therefore, findings were synthesized qualitatively.

### 2.7 Quality Assessment

The methodological quality and reporting transparency of the review were assessed using the Scale for the Assessment of Narrative Review Articles (SANRA) [21]. The SANRA framework guided the evaluation of scientific reasoning, literature coverage, referencing quality, and balanced interpretation of findings (Figure S1).

### 2.8 Ethical Considerations

This study was based entirely on previously published literature and did not involve direct human participation or primary data collection. Therefore, ethical approval and informed consent were not required. The review was conducted according to principles of academic integrity, transparency, and accurate reporting of evidence.

## 3 Biological Determinants

Biological determinants of BU include pathogen, host, and disease‐related factors that influence susceptibility, severity, and health outcomes [22]. The causative agent, *M. ulcerans*, produces the toxin mycolactone, which drives tissue necrosis and immune suppression, with strain differences and bacterial load affecting lesion progression [23]. Host characteristics, including age, sex, immune status, nutritional status, and genetic predisposition, also play a critical role in determining disease severity and the recovery process [24]. Disease‐specific factors, such as lesion type, size, and the timing of treatment initiation, further influence functional impairment, scarring, and long‐term outcomes [25] (Table 1; Figure 1).

**TABLE 1:** Biological determinants and their influence on Buruli ulcer outcomes in selected studies.

References	Country	Study design	Biological determinant	Influence on Buruli ulcer	Main findings
Fevereiro et al. (2019) [26]	NA	Review	Host immune response	Innate and adaptive immunity are critical in controlling *Mycobacterium ulcerans* infection; cytokine balance determines severity	Innate and adaptive immunity determine lesion formation, healing, and recurrence
Yotsu et al. (2025) [27]	NA	Review	Host immune response	Mycolactone suppresses immune cells, delaying lesion recognition and healing	Mycolactone‐induced immunosuppression leads to tissue necrosis and silent progression
Yotsu et al. (2024) [28]	Japan/Ghana	Review	Pathogen factors	Mycolactone‐mediated apoptosis, immune suppression, and painless ulcers	Mycolactone suppresses inflammation, causing ulcer progression
Fevereiro et al. (2019) [26]	NA	Review	Pathogen factors	Bacterial load and mycolactone persistence → tissue destruction	High bacterial load correlates with lesion size, depth, and severity
Tchatchouang et al. (2024) [29]	Cameroon	Systematic review	Pathogen factors	Geographic and strain variations influence virulence	Strain differences affect toxin production, disease outcomes
Osei‐Owusu et al. (2018) [12]	Ghana	Review	Lesion characteristics	Depth and undermined edges increase healing time, secondary infection risk	Deep lesions require longer recovery and a higher care burden.
Yotsu et al. (2025) [27]	Japan/Ghana	Review	Treatment timing and response	Early antibiotics reduce necrosis, accelerate healing, and delay complex lesions	Prompt rifampicin‐based therapy improves recovery and functional outcomes
Osei‐Owusu et al. (2018)[12]	Ghana	Review	Treatment timing and response	Delayed rural treatment → larger lesions, surgical intervention	Early detection critical for reducing functional impairment
Yotsu et al. (2024) [28]	Japan/Ghana	Review	Comorbidities, age, sex, nutrition	Comorbidities, age, sex, and nutrition affect lesion severity and healing	HIV, malnutrition, children, older adults → worse outcomes
Fevereiro et al. (2019) [26]	NA	Review	Comorbidities, age, sex, nutrition	Children → immature immunity; older adults → slower healing	Age and immune status critical determinants of progression
Fevereiro et al. (2019) [26]	NA	Review	Additional host factors	Genetics, prior skin trauma, and inflammatory markers affect severity	Host susceptibility, trauma, and cytokines IL‐1/TNF‐α linked to lesion progression
Yotsu et al. (2025) [27]	Japan/Ghana	Review	Additional host factors	Pharmacogenetics influences antibiotic metabolism	Genetic differences affect rifampicin/clarithromycin efficacy

**FIGURE 1:**
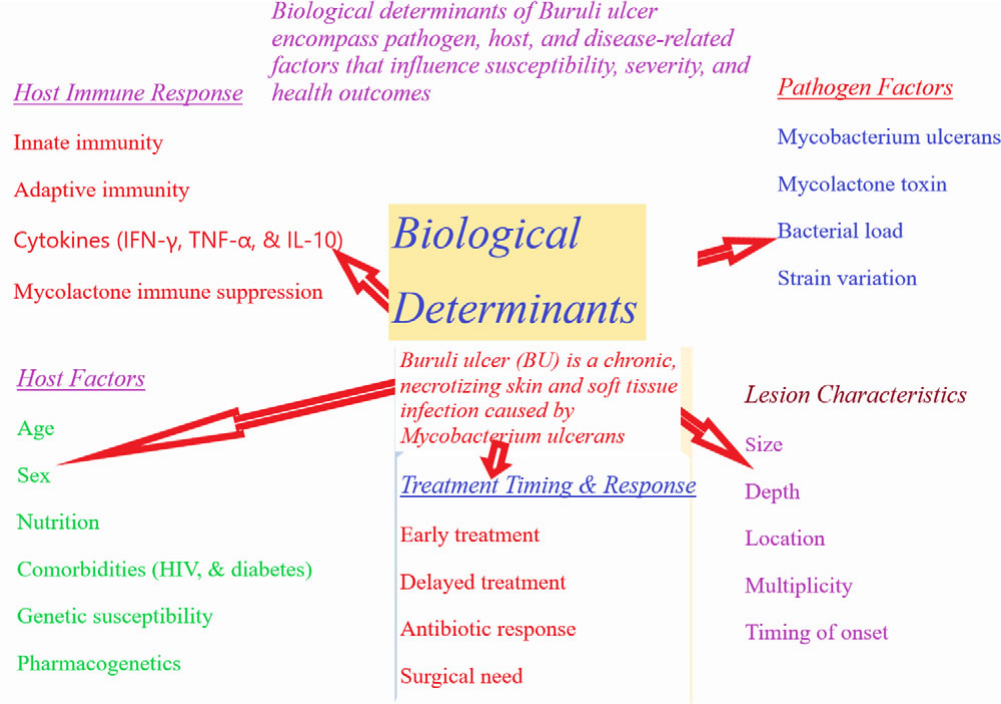
Biological determinants and their influence on Buruli ulcer.

### 3.1 Host Immune Response

The host immune response is a central determinant of BU susceptibility, progression, and clinical outcomes [23]. Both innate immunity, including macrophages, neutrophils, dendritic cells, and natural killer cells, and adaptive immunity, T‐cell and B‐cell‐mediated responses, play critical roles in controlling *M. ulcerans* infection [26]. Innate immune cells initially recognize the pathogen through pattern recognition receptors (PRRs) such as Toll‐like receptors, leading to cytokine and chemokine production, recruitment of inflammatory cells, and activation of antimicrobial mechanisms [30]. Recognition of *M. ulcerans*‐derived molecular patterns, including cell‐wall components, can activate NF‐*κ*B and other innate signaling pathways, promoting the production of inflammatory mediators that contribute to early host defense [31]. Dysregulated or insufficient innate responses can permit bacterial proliferation and enhance tissue necrosis [32]. However, the relatively weak inflammatory response characteristic of many BU lesions distinguishes *M. ulcerans* infection from several other mycobacterial diseases [2]. This weak inflammatory response is closely associated with the immunomodulatory activity of mycolactone.

Adaptive immunity, particularly Th1‐mediated cellular responses, is crucial for bacterial clearance. IFN‐γ and TNF‐α secreted by activated T cells stimulate macrophages to kill intracellular bacteria [33]. CD4+ T‐cell responses are particularly important for effective antimycobacterial immunity [34]. Th1‐associated IFN‐γ production contributes to pathogen control, whereas regulatory pathways involving IL‐10 can restrict excessive inflammation [35]. However, dominant IL‐10 activity may also impair effective pathogen control. Conversely, a skewed Th2 response or immunosuppressive cytokines (e.g., IL‐10) may limit effective bacterial clearance and contribute to chronic lesions [36]. Host immune responses are also influenced by age, sex, and nutritional status, with children, older adults, and malnourished individuals often exhibiting weaker immunity, delayed healing, and increased risk of recurrence [37, 38]. Mycolactone also interferes with intracellular protein synthesis and cellular signaling, thereby impairing the function and survival of immune cells and contributing to the characteristic hypo‐inflammatory phenotype of BU [39]. Genetic polymorphisms in immune‐related genes, such as cytokine receptors or HLA types, may further modulate susceptibility and severity [40]. The balance between effective antimicrobial immunity and toxin‐mediated immunosuppression may therefore help determine whether infection remains localized or progresses to extensive tissue destruction [41].

Fevereiro et al. (2019) described complementary roles for innate and adaptive immunity in BU lesion formation, healing, and recurrence [26]. These findings are consistent with evidence that effective cellular immunity contributes to bacterial control, whereas immune dysregulation may influence tissue damage and delayed healing. However, the relationship between immune activation and clinical outcome is not straightforward. The inflammatory response may contribute to bacterial control while also promoting tissue injury [34]. In contrast, the immunosuppressive effects of mycolactone may limit inflammation and permit persistent infection [23]. Thus, immune responses should not be interpreted simply as either protective or harmful. Yotsu and Phillips, (2025) further emphasized mycolactone‐mediated suppression of local immune responses, including effects on immune‐cell function and tissue repair [42]. These findings suggest that the clinical course of BU reflects a balance between pathogen‐mediated immune suppression and host‐mediated inflammatory responses. No single immune pathway appears to be dominant.

Evidence concerning differences in immune responses among patient groups is less consistent. Osei‐Owusu, (2018) reported altered immune responses and greater disease severity among children, older adults, and immunocompromised individuals, including patients with HIV or malnutrition [12]. However, these associations cannot be attributed solely to immune status. Age, nutritional status, co‐infections, healthcare access, and treatment delay may act as concurrent determinants. The available evidence therefore supports an association between host condition and disease severity. However, it remains insufficient to establish the independent contribution of specific immune abnormalities.

### 3.2 Pathogen Factors

*M. ulcerans* is a slow‐growing environmental mycobacterium whose pathogenicity is primarily mediated by mycolactone, a potent macrolide toxin [6]. Mycolactone is central to the disease's clinical manifestations: it induces apoptosis of host cells, suppresses immune responses, and causes extensive tissue necrosis [28]. By interfering with local inflammatory signaling, mycolactone allows the bacteria to proliferate with minimal host immune interference, resulting in painless yet progressive ulceration, which often delays healthcare‐seeking behavior [42]. Bacterial load also plays a main role in determining disease severity [6]. Higher concentrations of *M. ulcerans* correlate with larger, deeper, and more persistent lesions, increasing the risk of functional impairment and scarring [43].

Furthermore, variations in bacterial strains and toxin subtypes can influence virulence and the kinetics of disease progression [44]. Recent advances in molecular epidemiology, genomics, and whole‐genome sequencing (WGS) of *M. ulcerans* have improved characterization of genetic diversity, strain‐relatedness, geographic variation, and pathogen evolution [45]. These approaches can help investigate potential transmission patterns. They can also identify genetic differences associated with virulence. However, their application remains limited in many endemic African settings because of cost, laboratory infrastructure, and technical requirements [46]. For example, certain strains produce higher levels of mycolactone, leading to more aggressive tissue damage and slower healing [24].

High bacterial load and potent toxin activity may prolong antibiotic therapy or adjunctive surgical interventions [47]. Additionally, pathogen characteristics interact closely with host immune factors and social determinants, shaping clinical outcomes and the overall burden of BU [22]. Yotsu et al. (2024) [28] reported that *M. ulcerans* virulence is mainly mediated by mycolactone. These findings are consistent with experimental and clinical evidence linking mycolactone activity to immune suppression and tissue destruction, supporting a strong mechanistic basis for its role in disease severity. However, mycolactone‐mediated pathogenicity does not by itself establish how individuals acquire infection. The study strongly explains toxin‐mediated pathogenicity. However, transmission mechanisms remain insufficiently clarified.

Fevereiro et al. (2019) found that bacterial load and persistence of mycolactone in lesions determine tissue destruction and clinical severity [26]. This provides a complementary clinical perspective to the toxin‐centered mechanism described by Yotsu et al. (2024) [28]. It suggests that pathogen burden and sustained toxin activity may jointly influence lesion progression. Nevertheless, the available evidence does not establish a consistent quantitative relationship between bacterial load, mycolactone concentration, and clinical severity across patients.

Tchatchouang et al. (2024) demonstrated that geographic variation in *M. ulcerans* strains may influence toxin production, disease prevalence, and outcomes [29]. This observation broadens the interpretation beyond host and lesion‐level factors. It suggests that pathogen heterogeneity may contribute to differences between endemic settings. However, evidence directly linking specific strain characteristics to differences in human disease severity remains limited. The study highlights strain heterogeneity, although regional variability may limit generalizability. Yotsu et al. (2024) highlighted pathogen‐related factors, including the biological characteristics and persistence of *M. ulcerans* [28]. These factors may influence BU occurrence and disease development.

### 3.3 Lesion Characteristics

Lesion characteristics are major biological determinants in BU, as they reflect both host‐pathogen interaction and the stage of disease progression [48, 49]. The size of a lesion often correlates with disease severity, as larger lesions are associated with extensive tissue necrosis, higher bacterial burden, delayed healing, and increased risk of secondary infections [21]. Location of lesions also matters clinically and functionally [50]. Lesions on the limbs, particularly over joints, may limit mobility, increase the likelihood of contractures, and complicate surgical management. Lesions on the trunk or face, though less common, may carry additional cosmetic and psychosocial consequences [51]. Superficial lesions may heal with minimal scarring, whereas deep lesions involve subcutaneous tissue, tendons, or even bone, leading to functional impairment and prolonged recovery [49]. Multiplicity, or the presence of multiple lesions, often indicates systemic dissemination or delayed diagnosis, which can worsen prognosis and complicate treatment strategies [34].

The timing of onset, the interval between infection and clinical presentation, affects biological outcomes [52]. Early‐detected lesions typically respond better to antibiotics and surgical care, whereas delayed detection allows continued mycolactone‐mediated tissue destruction, increasing the risk of disability [53]. Lesion characteristics provide valuable prognostic information and guide individualized management plans, linking biological severity to functional and psychosocial consequences [27]. Osei‐Owusu et al. (2018) reported that deeper lesions with undermined edges delay healing and increase the risk of secondary infection [12]. This finding is clinically consistent. However, it may be limited by confounding factors, measurement variability in lesion assessment, and reduced generalizability due to a restricted study population. The association between lesion characteristics and delayed healing is also supported by studies showing greater lesion severity associated with prolonged recovery and functional impairment, suggesting a broadly consistent relationship. Nevertheless, differences in lesion classification, follow‐up duration, and definitions of healing make direct comparison between studies difficult. Thus, the evidence supports lesion severity as a clinically relevant prognostic factor, but the magnitude of its effect remains uncertain.

### 3.4 Treatment Timing and Response

The timing of treatment is a critical biological determinant in the management of BU [53]. Early initiation of antibiotic therapy, typically with rifampicin‐based regimens combined with clarithromycin or streptomycin, has been shown to reduce lesion progression, promote faster healing, and lower the risk of recurrence [47]. Prompt therapy limits the necrotizing effects of mycolactone, thereby preserving surrounding tissue and reducing the need for extensive surgical interventions [54]. Delayed treatment, on the other hand, often leads to larger, deeper, and more complex lesions, increasing the likelihood of functional impairment and long‐term disability [6]. Advanced lesions may require surgical excision, skin grafting, or debridement, which carry additional risks of infection, scarring, and prolonged recovery [51]. Moreover, delayed therapy can prolong the duration of mycolactone exposure, exacerbating immunosuppression and local tissue destruction [34].

Treatment response also varies among patients depending on host factors such as immune status, age, nutritional condition, and comorbidities like HIV infection [4]. These factors can influence antibiotic efficacy, wound healing, and susceptibility to secondary infections [52]. Yotsu et al. (2025) found that early antibiotic therapy, particularly rifampicin and clarithromycin, reduces tissue necrosis, accelerates healing, and prevents recurrence [27]. This finding is clinically relevant. Its consistency with evidence showing better outcomes with earlier treatment strengthens the conclusion that treatment timing is an important determinant of BU outcomes. However, the relative contribution of early treatment versus baseline lesion severity and differences in clinical care remains uncertain. Confounding factors and limited generalizability may, therefore, weaken the evidence. Osei‐Owusu et al. (2018) reported that delayed treatment in rural areas allows lesion progression, often requiring surgical intervention [12]. This finding complements Yotsu et al. (2025) [27] by linking delayed access to more advanced disease and greater treatment requirements. However, the relationship may not be entirely causal; rural residence can also reflect differences in healthcare availability, socioeconomic conditions, and disease recognition. Access‐related confounding and selection bias may influence the results.

### 3.5 Comorbidities, Age, Sex, and Nutritional Status

Comorbidities, age, sex, and nutritional status are critical factors influencing the susceptibility, progression, and outcomes of BU [55]. Comorbidities, such as HIV infection, diabetes, or other chronic illnesses, compromise host immunity and may lead to more severe lesions, delayed healing, and increased risk of secondary infections [6]. In HIV‐positive individuals, for instance, immune suppression can exacerbate the effects of *M. ulcerans* and prolong treatment response [26]. Children often have developing immune systems, which may alter their ability to contain infection; older adults may experience immunosenescence, leading to slower healing, increased lesion severity, and higher risk of complications [8]. Sex differences may also influence immune responses, with some studies suggesting variations in inflammatory cytokine profiles, healing rates, and hormonal modulation of immune function between males and females [34].

Nutritional status profoundly affects immune competence and tissue repair. Deficiencies in essential micronutrients, such as zinc, vitamin D, and iron, can impair wound healing, reduce the effectiveness of innate and adaptive immune responses, and increase susceptibility to infection [6, 56]. Yotsu et al. (2024) reported that comorbidities such as HIV, malnutrition, and chronic illness compromise immunity and increase susceptibility to severe BU lesions [28]. This is consistent with known immunopathological mechanisms. However, the study may be limited by confounding between comorbid conditions. Its observational design also restricts causal inference. Importantly, the available evidence does not establish whether individual comorbidities independently increase BU severity. Their effects may instead be mediated through nutritional status, immune dysfunction, delayed diagnosis, or limited access to care. Thus, differences between studies may partly reflect variation in the distribution and severity of comorbid conditions across populations.

Fevereiro et al. (2019) highlighted that children have immature immune systems, whereas older adults show slower healing rates [26]. However, the study may not fully account for environmental or healthcare access factors. Age‐related immune differences may also be overgeneralized across heterogeneous populations. These findings should therefore be interpreted cautiously. Age‐related differences in clinical outcomes may reflect biological variation as well as differences in exposure, caregiving, access to treatment, nutrition, and rehabilitation. Consequently, the evidence is insufficient to attribute poorer outcomes solely to age‐dependent immune function. Pratima et al. (2005) showed that males tend to have more trunk lesions. Females may experience delayed treatment due to caregiving roles [57]. However, the findings may be context‐specific. The older data may also reduce generalizability to current healthcare settings. The apparently different patterns by sex may reflect differences in occupation, exposure, mobility, caregiving responsibilities, healthcare‐seeking behavior, or local gender norms rather than intrinsic biological susceptibility alone. Direct comparisons across studies are also limited because lesion location and treatment delay may be measured differently.

### 3.6 Additional Host Factors

Beyond general immune response and lesion characteristics, several host‐specific biological determinants influence BU susceptibility, progression, and outcomes [58]. Genetic susceptibility plays a role, as polymorphisms in immune‐related genes may modulate the host's ability to contain *M. ulcerans* infection, influencing lesion severity and healing rates [59]. Skin integrity and prior trauma are important, as breaks in the skin barrier, whether from minor injuries, insect bites, or other wounds, facilitate bacterial entry and colonization [47]. Microbiome interactions are emerging as a significant determinant [60]. The composition of the skin microbiota can influence susceptibility to secondary infections, inflammation, and local immune responses, thereby affecting lesion progression and healing [6]. Systemic inflammatory markers, such as cytokines IL‐1 and TNF‐α, reflect the host's immune activation and are associated with disease severity, tissue necrosis, and the risk of complications [61]. Host pharmacogenetics affects the metabolism and efficacy of antibiotics commonly used to treat BU, including rifampicin and clarithromycin [62]. Genetic differences in drug‐metabolizing enzymes can impact therapeutic outcomes, treatment duration, and the likelihood of recurrence [12].

Fevereiro et al. (2019) reported that genetic susceptibility, prior skin trauma, and systemic inflammation influence the severity of BU [26]. They also found that IL‐1 and TNF‐α correlate with lesion progression. However, the evidence is observational, limiting causal inference. Inflammatory markers may reflect disease severity rather than predict progression. Thus, these findings should be interpreted as associations rather than evidence that inflammatory cytokines independently drive lesion progression. Differences in patient characteristics, disease stage, and timing of biomarker measurement may also contribute to variation between studies. Yotsu et al. (2025) highlighted that host pharmacogenetics can affect antibiotic metabolism and treatment efficacy 46]. However, the evidence is still emerging. It is based on limited early‐stage studies, with insufficient large‐scale validation linking genotypes to clinical outcomes. Compared with the more established evidence for host inflammatory responses, pharmacogenetic evidence remains considerably less developed. It is not yet clear whether genetic variation produces clinically meaningful differences in treatment response. Therefore, pharmacogenetic findings should currently be considered hypothesis‐generating rather than sufficient for treatment stratification.

## 4 Social Determinants

Social determinants play a critical role in BU outcomes, influencing both disease severity and HRQoL [11]. Poverty, limited education, low health literacy, geographic barriers, cultural beliefs, and restricted healthcare access can delay recognition and treatment, increasing advanced lesions and disability [63, 64]. Gender roles, household responsibilities, and traditional healing may further influence care‐seeking, whereas family and community support can improve adherence, recovery, and psychosocial well‐being [63] (Table 2; Figure 2).

**TABLE 2:** Social determinants and their influence on Buruli ulcer (BU) outcomes in selected studies.

References	Country/Region	Study design	Social determinants	Influence/Focus	Findings
Ackumey et al. (2012) [8]	Ghana (Amasaman, Ga West)	Semi‐structured EMIC interviews, 178 BU patients	Stigma and social perceptions	Examined socio‐cultural determinants of timely vs. delayed treatment	Median treatment: 25 days (pre‐ulcer), 204 days (ulcer). Income loss and herbalist use delayed treatment; health access encouraged timely treatment. Stigma and perceptions influenced health‐seeking behavior
Azubuike et al. (2025)[14]	Nigeria (Enugu State)	Qualitative, 1 FGD + 6 KIIs, BU patients	Mental health and HRQoL/Stigma and social perceptions	Explored the psychosocial experiences of BU patients using the socioecological model	Patients reported pain, disfigurement, social rejection, and isolation. Interpersonal and community‐level stigma affected reintegration. Highlighted the need for community awareness and support
Agbo et al. (2019) [17]	Benin (2 communes)	Ethnography, semi‐structured interviews, 45 women	Social support and community networks	Gendered impact on household health production and care decisions	Women are responsible for arranging care while maintaining household duties. Social support is critical for care; decentralization is preferred to avoid displacement
de Zeeuw et al. (2014)[65]	Ghana and Benin	Cross‐sectional, 143 former patients	Mental health and HRQoL/community reintegration	Participation restrictions post‐BU treatment	67/143 patients experienced restrictions. Predictors: female gender, perceived stigma, functional limitations, and larger lesions
Ahorlu et al. (2013) [66]	Ghana (Ga West and South)	Semi‐structured interviews, 33 patients + 7 caretakers	Economic burden and poverty/Social support	Factors influencing health‐seeking decisions	Family advice (42.5%) and health worker advice (45%) influenced care seeking. Financial considerations less influential, especially with free antibiotic treatment
Onwuka and Oparaocha, (2025) [9]	Nigeria (Imo State)	Mixed‐methods, 300 respondents + IDIs, KIIs, FGDs	Access to healthcare services/Stigma	Cultural beliefs and structural barriers to timely treatment	27.4% sought biomedical care within 2 weeks. Delays are linked to low education, spiritual causation beliefs, distance, and lack of insurance. A traditional healer uses common.De
Klís et al., (2014) [63]	Ghana	Cross‐sectional follow‐up, 127 former BU patients	Mental health and HRQoL	Long‐term quality of life after early antibiotic treatment	Median DLQI = 0; 85% reported no or a small effect on life. Good physical and psychological HRQoL. Early treatment leads to better outcomes
Fevereiro et al. (2020) [565]	Multi‐country (systematic review)	Systematic review and meta‐analysis, 29 studies	Gender, age, and cultural influences	Individual and clinical variables associated with BU acquisition	Age extremes are most affected; males among children. HIV infection and the SLC11A1 allele are associated with a higher prevalence. BCG is not protective
Popa et al. (2023)[67]	Sub‐Saharan Africa	Narrative review	Access to healthcare/Treatment	Strategies: Recent advances in BU management	Antibiotics: rifampicin, streptomycin, clarithromycin. Early detection crucial. Improved adherence strategies and wound care are recommended
Yotsu et al. (2025) [27]	Sub‐Saharan Africa	Narrative review	Access/Mental health and HRQoL/Community reintegration	Pathogenesis, diagnosis, treatment, and HRQoL	Mycolactone is central to tissue damage. PCR is gold standard, but it is limited in low‐resource settings. Treatment: oral rifampicin + clarithromycin; psychosocial and physiotherapy support is essential

Abbreviations: DLQI, Dermatology Life Quality Index; HRQoL, health‐related quality of life.

**FIGURE 2:**
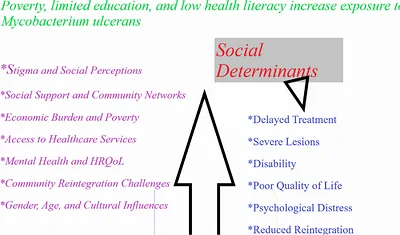
Biological determinants and their influence on Buruli ulcer. HRQoL, health‐related quality of life.

### 4.1 Stigma and Social Perceptions

Stigma is a major social determinant influencing the experiences of individuals affected by BU in Africa [16]. The disease, often characterized by visible skin lesions, deformities, and long‐term disabilities, can trigger fear, misconceptions, and negative attitudes within communities. Many affected individuals report being labeled as “contagious,” “cursed,” or “unproductive,” leading to social exclusion and isolation [14, 16]. These stigmatizing perceptions are reinforced by a lack of awareness about the etiology, transmission, and treatment of BU, contributing to delayed healthcare seeking and worsening disease outcomes. The consequences of stigma extend to education and employment. Children affected by BU may face absenteeism or drop out due to ridicule or bullying, whereas adults may lose employment opportunities or be marginalized in community activities [16]. Social participation is also curtailed, as patients often avoid public spaces or community gatherings to escape judgment or discrimination [10, 65]. This social withdrawal not only affects psychological well‐being but also limits the ability to access community support networks, reinforcing cycles of poverty and disease vulnerability.

Ackumey et al. (2012) reported that patients delayed treatment due to fear of social judgment and a preference for socially accepted herbalists [8]. The study offers strong contextual insight but is limited by self‐report bias and reduced generalizability. Azubuike et al. (2025) found that social rejection, loss of social roles, and isolation within families and peer networks contributed to emotional distress [14]. This recent study is relevant, but its cross‐sectional design limits causal interpretation. Its findings are consistent with evidence linking stigma and social exclusion to poorer psychosocial outcomes. However, differences in populations and outcome measures make direct comparison with other studies difficult. de Zeeuw et al. (2014) identified perceived stigma, larger lesions, and female gender as predictors of restricted social participation affecting education and employment [65]. The study provides useful predictor analysis, but confounding factors may not have been fully controlled. The association between larger lesions and restricted participation suggests that clinical severity and social consequences may reinforce one another. However, the relative contribution of stigma, lesion severity, and gender remains uncertain because these factors may be interrelated.

### 4.2 Social Support and Community Networks

Social support plays a critical role in the recovery and overall well‐being of persons affected by BU [17]. Family, peers, and broader community networks provide emotional, practical, and financial assistance that can significantly influence a patient's coping mechanisms [68]. Emotional support from family members and peers helps reduce psychological distress, anxiety, and feelings of isolation that often accompany chronic skin diseases like BU [69]. Practical support, such as assistance with transportation to health facilities, wound care, and daily activities, can improve adherence to treatment regimens and ensure timely follow‐up, which is essential to prevent complications and long‐term disability [11]. Community networks also shape health‐seeking behaviors. Individuals embedded in supportive communities are more likely to access biomedical care early, with prescribed antibiotic therapy, and participate in rehabilitation programs [14]. Conversely, weak social support or social exclusion may delay treatment, worsen clinical outcomes, and exacerbate stigma.

Social capital, the resources and trust embedded within a community, further determines the effectiveness of these networks [70]. Communities with strong cohesion and collective action can mobilize resources, reduce stigma, and create environments that foster reintegration of BU patients post‐treatment [10, 11, 69]. Agbo et al. (2019) [17] found that women managed household care while attending hospital visits. Their ability to mobilize kin support influenced treatment decisions and household welfare [17]. However, contextual and sampling bias may limit generalizability and causal inference. The study highlights the importance of family support in enabling women to balance treatment and household responsibilities. However, its observational design does not establish whether kin support directly improved treatment decisions or household welfare.

Azubuike et al. (2024) reported that supportive family and community networks reduced psychosocial stress. In contrast, strained relationships increased feelings of isolation [120]. However, reliance on self‐reported data may introduce bias, and causal direction remains unclear. The findings indicate that the quality of social relationships may be more important than their mere presence, linking supportive networks with better psychosocial outcomes. However, the cross‐sectional evidence cannot determine whether poor social support causes psychological distress or whether distress contributes to strained relationships. Ackumey et al. (2012) showed that encouragement from relatives and health services promoted timely treatment [8]. However, contextual and temporal limitations may reduce current applicability. This finding extends the evidence by suggesting that social support may influence not only psychosocial well‐being but also the timing of healthcare‐seeking. Nevertheless, the relative contribution of family encouragement compared with health‐service support was not clearly established, limiting conclusions about which source of support is most influential.

### 4.3 Economic Burden and Poverty

BU disproportionately affects populations in low‐resource settings, where poverty is both a risk factor and a consequence of the disease [71]. The direct costs of BU treatment include medical fees for hospitalization, medications, wound dressings, and surgical interventions for severe lesions [72]. These costs are often unaffordable for affected families, leading to delayed care‐seeking or reliance on alternative remedies, which can worsen disease severity [73]. Indirect costs further exacerbate the economic burden. Many patients must travel long distances to reach health facilities, incurring transportation and accommodation expenses [72]. Caregivers, often family members, may lose income due to time spent accompanying patients, reducing household earnings and productivity. Additionally, prolonged treatment and disability may limit the patient's ability to work, contributing to long‐term financial insecurity [73]. Poverty also influences disease progression and rehabilitation outcomes [70]. Individuals from economically disadvantaged households are more likely to present with advanced lesions, increasing the risk of complications and extended hospitalization. Limited financial resources may hinder access to physiotherapy, nutritional support, or assistive devices, which are critical for recovery and community reintegration [72].

The cyclical relationship between poverty and BU highlights the need for interventions that provide financial support, subsidized care, and community‐based programs to reduce the economic impact and improve health outcomes [71]. Although BU treatment is often free, indirect costs, such as transportation, lost income, and caregiving responsibilities, remain substantial barriers. Ackumey et al. (2012) found that income loss was negatively associated with timely treatment [8]. This finding suggests that the economic consequences of BU may themselves contribute to treatment delay. However, because income loss may also reflect disease severity, caregiving demands, or distance from healthcare facilities, the independent effect of income loss is difficult to establish. The study was cross‐sectional, so it cannot establish causality between income loss and delayed treatment‐seeking. Ahorlu et al. (2013) noted that financial considerations [66] still influenced health‐seeking behavior, even after the introduction of free antibiotics. This finding indicates that removing direct treatment costs may not eliminate economic barriers to care, particularly when transportation, food, accommodation, and income loss remain relevant. Compared with Ackumey et al. (2012) [8], the study therefore broadens the economic explanation beyond income loss to include the wider costs of accessing care. This may reflect self‐reported financial perceptions and unmeasured residual barriers. The study context may also limit generalizability to other healthcare settings.

Onwuka and Oparaocha, (2025) reported that poverty delayed early care‐seeking and contributed to more severe disease outcomes [9]. This finding is consistent with earlier evidence linking economic disadvantage with delayed treatment, but it provides a broader association between poverty, care‐seeking, and disease severity. However, the relationship may be partly explained by correlated factors such as health literacy, geographic accessibility, and availability of healthcare services. Although the study provides recent evidence, it is still limited by its observational design. Therefore, the reported association should not be interpreted as evidence that poverty independently causes delayed treatment or greater disease severity. Ahorlu et al. (2013) emphasized that family and peer advice strongly influenced treatment‐seeking behavior, often outweighing financial considerations [746]. The study offers valuable insights into social influence, but it may be limited by sampling bias and limited quantification of determinants.

### 4.4 Access to Healthcare Services

Access to healthcare is a critical determinant of disease outcomes in BU, influencing both the severity at presentation and the long‐term quality of life of affected individuals [11, 63]. The quality of available healthcare also plays a significant role. Limited training of healthcare personnel about BU identification and management can lead to misdiagnosis or inappropriate interventions [9]. Furthermore, the reliance on traditional medicine remains widespread in many communities. Although traditional healers provide culturally acceptable care, delays caused by initial treatment outside formal health systems often allow lesions to worsen, increasing morbidity and risk of disability [75, 76]. Ackumey et al. (2012) [8] found that proximity to health facilities and perceived availability of services improved timely treatment, whereas reliance on herbalists delayed care. However, the cross‐sectional design and reliance on recalled experiences limit causal interpretation, and the influence of cultural beliefs may not have been fully captured. Onwuka and Oparaocha, (2025) [9] reported that living >5 km from facilities and lack of health insurance were associated with delayed biomedical care. However, these findings provide more recent evidence; selection bias, residual confounding, and the cross‐sectional design limit the strength of the associations. Popa et al. (2023) [67] and Yotsu et al. (2025) [27] showed that limited diagnostic capacity, high laboratory costs, and rural resource constraints delay early detection and antibiotic use. However, their findings are largely context‐specific and may not be generalizable to settings with different healthcare infrastructure and financing systems.

### 4.5 Mental Health and HRQoL

BU patients often experience psychological distress, including depression and anxiety, due to the disfiguring nature of lesions, prolonged treatment, and social stigma associated with the disease [10]. Studies in Ghana, Benin, and Nigeria indicate that individuals with active BU show higher prevalence of depression (up to 27%) and anxiety (up to 42%) compared to healthy controls, highlighting the emotional burden of the disease [11]. Beyond active infection, even patients healed from BU may face long‐term psychosocial challenges [17]. Persistent scars, functional limitations, and reduced mobility can negatively impact physical and emotional well‐being. HRQoL assessments using instruments such as the World Health Organization Quality of Life‐BREF (WHOQOL‐BREF) and Dermatology Life Quality Index (DLQI) reveal lower scores in physical functioning, psychological well‐being, and social participation among former patients [11]. Effective management of BU, therefore, requires a holistic approach that addresses both physical healing and psychological recovery [6]. Early diagnosis, patient education, counseling, and structured rehabilitation programs are critical to improving long‐term HRQoL, reducing mental health sequelae, and supporting community reintegration [27]. This underscores the importance of integrating mental health services into BU care programs, particularly in endemic regions with high disease burden.

Azubuike et al. (2025) identified persistent depression, anxiety, and emotional distress among patients, compounded by stigma and social isolation [14]. The study highlights important psychosocial outcomes; however, limited methodological detail reduces confidence in its validity and generalizability. These findings are broadly consistent with evidence linking stigma, social isolation, and disease‐related disability to poorer psychosocial outcomes. However, differences in populations, measurement instruments, and study settings make direct comparisons difficult. de Zeeuw et al. (2014) showed that functional limitations and lesion severity directly influenced long‐term HRQoL, particularly in females and older patients [65]. The study provides strong evidence of an association between disease severity and HRQoL; however, its observational design and potential confounders limit causal inference. Compared with Azubuike et al. (2025) [14], this study provides stronger evidence for the relationship between clinical severity and HRQoL. However, it does not establish whether psychosocial factors independently contribute to these outcomes. Klís et al. (2014), in a follow‐up of the BU Randomized Clinical Trial (BURULICO), reported that patients with small lesions who received early antibiotic treatment had good long‐term HRQoL [63]. This trial provides evidence supporting early treatment benefits. However, attrition bias and selective patient characteristics may reduce external validity.

### 4.6 Community Reintegration Challenges

Individuals who have recovered from BU often face significant barriers attempting to reintegrate into their communities [68]. Despite successful medical treatment, the physical sequelae, such as scarring, contractures, or limb deformities, can limit mobility and functionality, making it difficult for patients to return to work, school, or daily social activities [6]. For children and adolescents, prolonged absence from school due to hospitalization or rehabilitation can result in academic setbacks, social isolation, and reduced peer interaction, which can negatively affect long‐term educational outcomes [77]. Persistent stigma remains a major obstacle. Even after healing, individuals may be labeled “diseased” or considered contagious, leading to exclusion from social events, community gatherings, and workplace opportunities [74]. This social marginalization can exacerbate psychological distress, fostering anxiety, depression, and reduced self‐esteem, and in turn limiting full participation in communal life [10].

Disability‐related barriers, including limited accessibility to public spaces, transport, or workplaces, further hinder reintegration [78]. In rural African settings, where BU is endemic, these challenges are compounded by limited rehabilitation services, lack of assistive devices, and inadequate community awareness programs [74]. de Zeeuw et al. (2014) reported persistent employment and participation restrictions due to disability and stigma [65]. However, unmeasured confounding and limited socioeconomic diversity may reduce generalizability. These findings indicate that physical disability and social stigma may jointly restrict reintegration, although the observational design limits conclusions about causality.

Azubuike et al. (2024) found that reintegration barriers are driven by community misconceptions and social isolation [10]. The study may be affected by contextual and self‐report biases. Its findings are broadly consistent with de Zeeuw et al. (2014) [65], supporting the importance of stigma and social exclusion. However, differences in study context and outcome assessment make direct comparison difficult. Agbo et al. (2019) showed that decentralized treatment improves reintegration through community‐based care [17]. However, selection bias and lack of long‐term follow‐up may limit the strength of conclusions. Unlike studies emphasizing barriers to reintegration, this study suggests that the organization and accessibility of care may modify these outcomes. However, methodological limitations prevent firm conclusions about the long‐term effectiveness of decentralized care.

### 4.7 Gender, Age, and Cultural Influences

Gender, age, and cultural factors play a pivotal role in shaping vulnerability, stigma, and access to care for individuals affected by BU [9, 16]. Gender norms in many African communities influence health‐seeking behaviors, caregiving roles, and social expectations [16]. Women often face additional barriers to accessing healthcare due to household responsibilities, limited financial autonomy, or societal restrictions, which can delay diagnosis and treatment. Men, on the other hand, may experience pressure to continue working despite illness, risking complications or prolonged disease severity [10]. Age is another critical determinant. Children with BU are particularly vulnerable due to dependency on caregivers for early recognition, transport to healthcare facilities, and adherence to lengthy treatment regimens [47]. Adolescents and young adults may face social exclusion, school absenteeism, or dropout from education, which can affect long‐term psychosocial outcomes [8]. Older adults may have comorbidities or mobility limitations, which make treatment and rehabilitation more challenging [12, 27].

Cultural beliefs and local practices also influence disease perception and care pathways. Misconceptions about the causes of BU, reliance on traditional medicine, and community myths can contribute to stigma, social isolation, and delayed healthcare seeking [79]. Cultural interpretations of disability or scarring may further compound mental health challenges and hinder community reintegration [10, 16, 79, 80]. Fevereiro et al. (2020) identified age extremes as more susceptible, with male preponderance in children [55]. However, this finding should be interpreted cautiously because age‐ and sex‐related differences may vary across populations. These differences may reflect variations in exposure, healthcare‐seeking, or underlying vulnerability rather than biological susceptibility alone. Agbo et al. (2019) emphasized the gendered nature of care, showing that women disproportionately bear household responsibilities and related vulnerabilities [17]. Although this finding supports a role for gender in shaping healthcare access, it contrasts with settings where gender differences in presentation or treatment‐seeking are less pronounced. This suggests that the effects of gender may be context‐dependent. The study provides important gender insights, but its generalizability may be limited by sampling bias.

Onwuka and Oparaocha, (2025) highlighted cultural beliefs, such as spiritual causation, delaying biomedical care‐seeking [9]. This finding is consistent with evidence linking cultural beliefs to delayed presentation. However, the extent of their influence may vary depending on local healthcare access, community norms, and trust in biomedical services. Because the evidence is largely observational and self‐reported, the independent contribution of cultural beliefs is difficult to establish. This study offers valuable behavioral insight, though self‐report bias and limited causal inference may affect its validity. Yotsu et al., (2024) reported that clinical outcomes, including lesion severity, vary by age and sex, suggesting interactions between biological and social factors [28]. This supports the possibility that demographic characteristics modify clinical outcomes. However, differences in study populations, outcome definitions, and adjustment for confounding factors make direct comparison with other studies difficult.

### 4.8 Diagnostic Challenges

Diagnostic challenges in resource‐limited African settings are strongly influenced by social and health‐system determinants [2]. Long travel distances, limited access to healthcare, diagnostic costs, weak referral and specimen‐transport systems, and shortages of trained personnel can delay diagnosis [81]. Low health literacy, limited awareness of early lesions, socioeconomic constraints, and reliance on traditional healers may further delay presentation and diagnostic confirmation [9]. These barriers can limit timely access to available diagnostic methods and contribute to delayed treatment. Strengthening community awareness, decentralized diagnostic services, referral and transport systems, and health‐care worker capacity could improve timely diagnosis and treatment [82].

### 4.9 Challenges in Treatment Adherence, Rehabilitation, and Long‐Term Disability

Although early antibiotic treatment improves healing and reduces complications, adherence may be affected by prolonged treatment, travel distance, treatment costs, adverse effects, limited health‐system support, and socioeconomic constraints [1, 83]. Access to physiotherapy, wound care, reconstructive services, assistive devices, and long‐term follow‐up remains limited in many endemic settings [27]. Severe lesions, delayed treatment, and inadequate rehabilitation can result in persistent functional limitations affecting daily activities, education, employment, and social participation [14].

## 5 Environmental Determinants and Transmission Ecology

Environmental conditions may influence BU distribution by shaping the ecological niches of *M. ulcerans* and patterns of human exposure [15] (Figure 3). Associations have been reported with wetlands, stagnant or slow‐moving water bodies, vegetation, and other aquatic environments, particularly in endemic areas in Africa [2]. However, the detection of *M. ulcerans* or mycolactone‐producing strains in environmental samples does not establish that these habitats are direct sources of human infection [20]. Environmental exposures, including proximity to endemic water bodies and contact with contaminated soil or water, are biologically relevant; they determine infection risk [9, 13]. Environmental disturbance may alter ecological conditions that favor bacterial persistence or human–environment contact [84].

**FIGURE 3:**
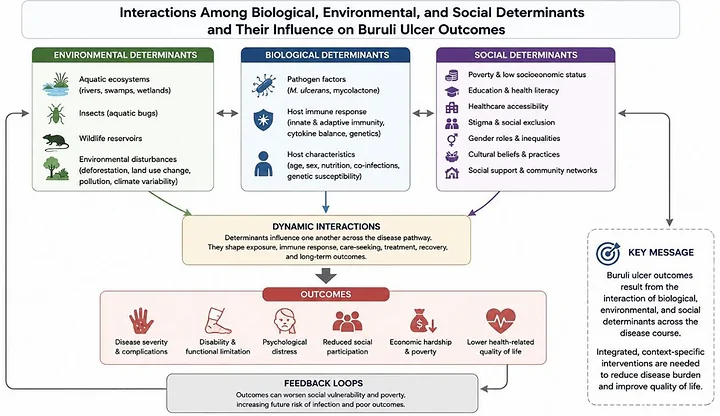
Interactions between biological, environmental, and social determinants.

Insects and other aquatic organisms have also been investigated as potential ecological contributors [48]. Climate variability and environmental disturbance may further influence the geographic distribution of BU by altering aquatic habitats and human exposure patterns [85]. Recent ecological and spatial studies suggest that variations in rainfall, flooding, temperature, water availability, and proximity to aquatic environments may be associated with differences in disease occurrence across endemic settings [86]. Land‐use changes, including deforestation, agricultural expansion, irrigation, and dam construction, may also modify aquatic ecosystems and human–environment interactions, potentially changing exposure patterns [15, 85].

Gohoho et al. (2025) found that environmental exposures, including proximity to water bodies and lack of protective clothing, increase the occurrence of lesions [52]. Tchatchouang et al. (2024) [29] highlighted geographic and environmental variation in *M. ulcerans* strains. This suggests that pathogen heterogeneity and local ecological conditions may contribute to differences in disease occurrence and virulence across endemic areas. Similarly, Yotsu et al. (2024)[40] described aquatic and animal reservoirs as components of the environmental ecology of *M. ulcerans*. As a review, the study provides a broad synthesis of ecological evidence but cannot independently establish causality. BU outcomes reflect the interplay of biological, environmental, and social determinants, including pathogen virulence, aquatic exposure, poverty, healthcare access, stigma, and cultural beliefs.

## 6 Integration of Biological and Social Determinants

Integrating biological and social determinants provides a holistic understanding of BU [8]. For example, severe infections may be more prevalent in marginalized populations, where delayed treatment and poor nutrition interact with the pathogen's virulence [86, 87] (Figure 4). Importantly, biological determinants interact with social factors, such as access to healthcare, stigma, and socioeconomic conditions, ultimately shaping the physical, psychological, and quality‐of‐life outcomes for individuals affected by BU in endemic regions [10, 14]. Although biological determinants, such as pathogen virulence, mycolactone production, lesion size, and infection severity, directly drive tissue damage and functional impairment, social determinants profoundly influence disease outcomes by affecting exposure, diagnosis, and access to care [6].

**FIGURE 4:**
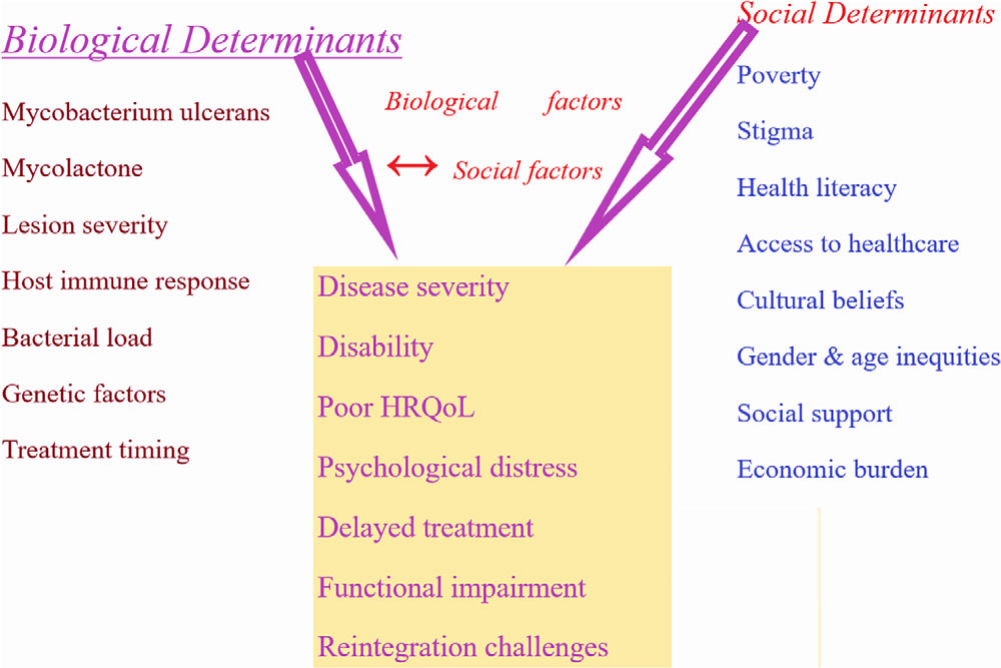
Biological and social determinants and their influence on Buruli ulcer. HRQoL, health‐related quality of life.

Delayed treatment is a key point of interaction as individuals from lower socioeconomic backgrounds often face financial constraints, limited access to healthcare facilities, or reliance on traditional medicine, resulting in prolonged exposure to the pathogen [8]. This delay exacerbates lesion severity, increases the risk of secondary infections, and elevates the likelihood of permanent disabilities [8, 68, 88]. Onwuka and Oparaocha, (2025) [9] show that stigma, social isolation, and spiritual beliefs contribute to anxiety, depression, and poorer HRQoL. This provides important psychosocial insight. However, reliance on self‐reported measures may introduce reporting bias. Cultural differences may also affect comparability. These findings complement the biological evidence from Popa et al. (2023) [67] and Jain and Yotsu (2024) [88] by indicating that BU outcomes cannot be explained by lesion biology alone. However, the available evidence does not establish whether psychosocial factors independently affect HRQoL. They may primarily mediate the effects of disease severity and disability.

## 7 Strength of Evidence and Emerging Mechanisms

The available evidence varies in strength. Established findings should be distinguished from mechanisms that remain under investigation [3]. The roles of mycolactone‐mediated immunosuppression and tissue damage, lesion severity, delayed diagnosis, and host immune responses in BU progression are supported by clinical and experimental evidence [34, 35, 55]. Similarly, socioeconomic barriers, stigma, healthcare access, and care‐seeking practices are consistently associated with delayed treatment and poorer functional or psychosocial outcomes [66, 70, 76, 79]. In contrast, proposed effects of specific differences in bacterial strain, pharmacogenetic variation, skin microbiome composition, micronutrient deficiencies, and some host genetic factors remain less firmly established [34, 53, 55, 77]. These should be considered emerging or hypothesis‐generating evidence. Environmental and geographic associations may indicate potential exposure pathways. Differences in study design, sample size, outcome measurement, and evidence type further limit causal inference [89].

## 8 Cross‐Country Variations in Buruli Ulcer Determinants and Health Outcomes

Evidence from African settings indicates both regional similarities and important country‐level differences in BU epidemiology, healthcare access, and social determinants [3]. In Ghana, delayed treatment has been associated with income loss, reliance on traditional healers, and limited healthcare access [70], whereas community and health‐worker support can facilitate earlier care‐seeking. In Nigeria, low education, spiritual beliefs, distance to healthcare facilities, and lack of insurance contribute to delayed biomedical care [76]. In Benin, gender roles, household responsibilities, and family support influence care‐seeking and treatment decisions [71]. Studies from Ghana and Benin also indicate that stigma and functional limitations can restrict social participation after treatment [66]. Across sub‐Saharan Africa, BU predominantly affects rural and underserved populations [77, 78, 79]. These findings suggest that disease‐control strategies should combine common regional priorities, such as early detection, accessible treatment, and stigma reduction, with context‐specific interventions addressing local barriers to diagnosis, treatment, rehabilitation, and social reintegration.

## 9 Policy and Practice Implications

Medical interventions should continue to focus on early diagnosis, antimicrobial therapy, and rehabilitation to reduce physical disability [27]. However, these must be complemented by psychosocial and community‐based interventions that mitigate stigma, promote mental health, and improve social reintegration of affected individuals. Health systems policies should incorporate integrated care models in which multidisciplinary teams, including clinicians, psychologists, social workers, and community health volunteers, are adequately trained and resourced [9]. Community engagement programs can increase awareness of BU early detection and reduce cultural barriers to care. Social protection policies, such as financial support for transportation, rehabilitation services, or vocational training, can alleviate the socioeconomic burden of BU [19]. At the programmatic level, policies should support monitoring and evaluation frameworks that track clinical outcomes and HRQoL, ensuring interventions target both physical and social dimensions [17]. Integrating biomedical and social strategies in policy and practice will enhance patient outcomes, reduce stigma, and improve long‐term quality of life for BU patients across Africa [9, 17] (Figure 5).

**FIGURE 5:**
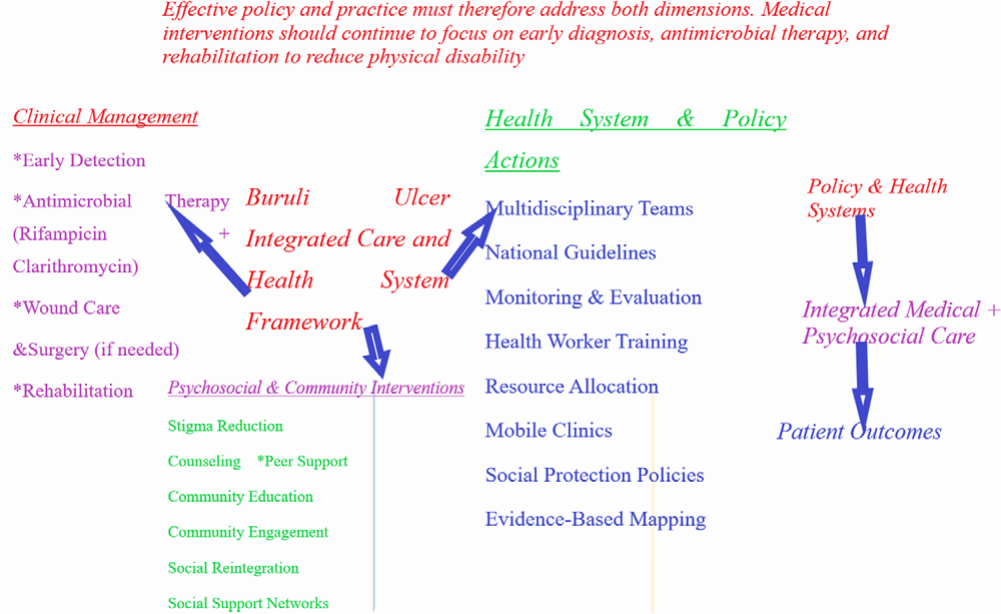
Integrated framework and healthcare system for Buruli ulcer.

### 9.1 Integrated Medical and Psychosocial Care

Effective management of BU requires a dual focus on both clinical treatment and psychosocial well‐being [11]. Although antimicrobial therapy and surgical interventions address the underlying infection and physical complications, patients often face significant mental health challenges, including anxiety, depression, and social stigma due to visible lesions or functional limitations. Integrating psychosocial care into medical treatment ensures that patients not only recover physically but also maintain psychological resilience and quality of life [68]. Addressing stigma is critical, as negative societal perceptions can delay treatment‐seeking, reduce adherence to therapy, and exacerbate social isolation [89, 90]. Interventions such as counseling, peer support groups, and community education programs help mitigate these effects by providing patients with coping strategies and reinforcing social acceptance [74]. Coordinated care models that involve physicians, nurses, mental health professionals, and social workers enable a comprehensive approach, allowing for simultaneous wound care, rehabilitation, and mental health support [27]. This integrated strategy is particularly important in endemic regions of Africa, where healthcare resources are limited, and patients may face economic, cultural, and geographic barriers to accessing care [10]. By combining biomedical treatment with psychosocial interventions, health systems can reduce disease burden, improve adherence, and promote long‐term recovery and reintegration into the community [11].

### 9.2 Community‐Based Interventions

Community‐based interventions are critical in managing BU, especially in endemic regions of Africa, where limited healthcare infrastructure, low health literacy, and cultural beliefs often delay treatment [9]. Engaging local leaders, traditional healers, and community health workers is essential to improving awareness about the disease, its early symptoms, and the importance of timely biomedical care [8]. Local leaders can act as trusted advocates, helping to reduce stigma and encouraging affected individuals to seek treatment rather than relying solely on traditional remedies, which may delay healing or worsen disease severity [76]. Promoting culturally appropriate education and outreach programs ensures that messages are relevant and understandable to diverse populations [82]. This includes using local languages, culturally familiar metaphors, and visual aids to explain BU prevention, early recognition, and care pathways. School‐based health education, radio campaigns, and community workshops can reach broader audiences, fostering a community‐wide understanding of disease transmission, the benefits of early intervention, and the role of hygiene and environmental measures in reducing risk [91, 92].

Effective community‐based interventions also emphasize participatory approaches, where community members are involved in planning, implementing, and monitoring programs [9]. This not only improves the uptake of health interventions but also empowers communities to sustain disease prevention efforts over time [8]. Ultimately, these strategies can significantly reduce the prevalence and severity of BU, improve HRQoL, and promote social reintegration of affected individuals. Community engagement has proven crucial in improving BU outcomes [92]. Ackumey et al. (2011) demonstrated through the BU Prevention and Treatment (BUPaT) program in Ghana that collaboration with local health workers, health education campaigns, and surveillance improved early reporting and treatment adherence [93]. Hausermann, (15) observed that rural populations often use hybrid treatment practices, including traditional healers [94]. This highlights the importance of respecting local knowledge and integrating community leaders. Muhi et al. further emphasized that involving community members in research design, such as controlled human infection models, improves transparency, acceptance, and culturally appropriate messaging [53].

### 9.3 Early Detection and Timely Treatment

Early detection of BU is critical to reducing morbidity, preventing severe tissue damage, and improving overall HRQoL [11]. Screening programs in endemic regions are essential, particularly in rural and resource‐limited settings where delayed presentation is common [63]. Community‐based surveillance strategies, such as training local health workers to recognize early skin lesions and encouraging self‐reporting, have been shown to facilitate prompt diagnosis [10]. Timely treatment, typically with a combination of antibiotics (rifampicin and clarithromycin), significantly reduces lesion size, prevents complications such as contractures and amputations, and decreases the need for surgical interventions [62]. However, treatment adherence may be affected by prolonged treatment, travel distances, treatment costs, limited health‐system support, adverse effects, and socioeconomic constraints [8]. These barriers may be particularly important in rural and underserved communities. Early intervention also limits the social and psychological consequences associated with visible scars, disability, and stigma, thereby improving mental health outcomes and social reintegration [4].

Barriers to early detection include limited awareness, cultural misconceptions, lack of access to healthcare facilities, and socioeconomic constraints [54]. Addressing these barriers requires health education campaigns, community engagement, and mobile outreach programs to identify cases before lesions progress. Integrating biomedical approaches with social support mechanisms ensures that individuals seek care early and adhere to treatment regimens, ultimately reducing disease burden and improving functional recovery [66]. Early detection is critical to reduce disease severity and long‐term disability [95]. BUPaT program [93] demonstrated that prompt antibiotic treatment prevented recurrences and reduced complications.

### 9.4 Public Health Policy and Integrated Disease Control Strategies

Public health policies should prioritize decentralization of diagnostic and treatment services, particularly in rural and underserved communities, to reduce geographic and financial barriers to care [9]. At the health‐system level, BU should be incorporated into NTD, primary healthcare, disability, and community health programs where appropriate [95]. Surveillance systems should capture not only case detection and treatment outcomes but also disability, treatment delay, and relevant social and psychosocial indicators [27]. Strengthening referral, reporting, and information systems can improve coordination across levels of care and support evidence‐based allocation of resources. Effective implementation also requires appropriate resource allocation and sustained health‐system capacity, particularly in settings where financial, infrastructural, and workforce constraints limit access to comprehensive BU services. Monitoring and evaluation frameworks should therefore assess both program performance and patient‐centered outcomes, allowing policies to be adapted according to emerging evidence and local needs.

## 10 Research Gaps and Unanswered Questions

Despite recognition of the multifactorial determinants of BU, important uncertainties remain regarding how biological, environmental, and social factors interact across the disease course. Most studies examine these domains separately, limiting understanding of their combined effects on disease progression, treatment adherence, disability, and HRQoL. The relative contributions of lesion severity, host responses, environmental exposure, treatment timing, socioeconomic conditions, stigma, and healthcare access remain insufficiently characterized. These contributions may differ across African settings. Long‐term outcomes after clinical cure are also poorly understood. In particular, evidence remains limited on the persistence of disability, restrictions in social participation, psychological well‐being, and HRQoL, especially among children and other vulnerable populations. Evidence is also insufficient to determine which combinations of medical, rehabilitative, psychosocial, community‐based, and health‐system interventions provide sustained benefits.

## 11 Future Directions

Future studies should use longitudinal, prospective, and mixed‐method approaches to evaluate determinants across the disease course and clarify temporal relationships between exposures and outcomes. Multiregional comparative studies should examine how biological susceptibility, environmental exposure, treatment timing, and social vulnerability jointly influence disease severity, disability, and HRQoL. Research should adopt standardized measures of stigma, socioeconomic conditions, healthcare access, psychological well‐being, functional status, and HRQoL to improve comparability across settings. Intervention studies should evaluate context‐specific integrated care packages. They should determine their effectiveness, feasibility, sustainability, and cost‐effectiveness over extended follow‐up. Particular attention should be given to identifying modifiable determinants that can be addressed at patient, household, community, and health‐system levels.

## 12 Limitations

This narrative review is limited by substantial heterogeneity in study designs, populations, settings, and outcome measures. The evidence base includes cross‐sectional, case‐control, ethnographic, and narrative studies. These differ in methodological rigor and may limit causal interpretation and direct comparison across studies. Variation in the assessment of stigma, HRQoL, psychosocial outcomes, functional status, and healthcare access further limits comparability. Vulnerable populations are also underrepresented in some studies. Geographic, socioeconomic, and cultural differences may affect the transferability of findings across African settings. Evidence evaluating integrated interventions remains limited, restricting conclusions regarding their comparative effectiveness and long‐term impact.

## 13 Conclusion

This review demonstrates that BU outcomes reflect interconnected biological, environmental, and social determinants rather than a single causal pathway. Pathogen characteristics, mycolactone activity, lesion features, and host factors contribute to disease severity and functional impairment. Environmental conditions may influence exposure and geographic distribution. Social conditions, including poverty, stigma, gender roles, cultural beliefs, education, healthcare access, and reliance on traditional healers, can influence diagnosis, treatment, adherence, and longer‐term well‐being. The overall evidence supports a comprehensive approach combining timely diagnosis and treatment with rehabilitation, psychosocial care, community engagement, and appropriate health‐system support. Recognizing the interaction among these determinants is essential for developing equitable and context‐sensitive strategies. Future research should prioritize longitudinal and multidisciplinary studies, standardized patient‐centered outcomes, and evaluation of integrated interventions targeting modifiable determinants. These priorities can help translate evidence into equitable, context‐sensitive strategies that improve BU outcomes and long‐term well‐being.

## Author Contributions

**Gudisa Bereda**: conceptualization, visualization, methodology, data curation, investigation, validation, project administration, supervision, resources, writing – original draft, writing – review and editing. The author checked and confirmed the final version of the manuscript.

## Funding

The author has nothing to report.

## Conflicts of Interest

The author declares no conflicts of interest.

## Supporting information




**Supplementary Figure 1**: SANRA—a scale for the quality assessment of narrative review articles.

## Data Availability

No data were used for the research described in the article.
