# Supplementary material for: Biological, Environmental, and Social Determinants of Buruli Ulcer in Africa: Their Interplay Across Disease Severity and Patient Outcome
Source: Public Health Chall. 2026 Oct 4;5(4):e70389. doi: 10.1002/puh2.70389 (PMC13634593; doi:10.1002/puh2.70389)
Supplement: Supplementary file 1 [file PUH2-5-e70389-s001.docx]

Supplementary Figure 1: SANRA—a scale for the quality assessment of narrative review articles

1) Justification of the article’s importance for the readership

The importance is not justified ______________________________________________________0

2

The importance is alluded to, but not explicitly justified___________________________________1

The importance is explicitly justified___________________________________________________2

2) Statement of concrete aims or formulations of questions

No aims or questions are formulated________________________________________________0

2

Aims are formulated generally but not concretely or in items of clear questions _____________1

One or more concrete aims or questions are formulated ________________________________2

3) Description of the literature search

The search strategy is not presented ________________________________________________0

2

The literature search is described briefly _____________________________________________1

The literature search is described in detail, including search terms and inclusion criteria _______2

4) Referencing

Key statements are not supported by references _______________________________________0

2

The referencing of key statements is inconsistent ______________________________________1

Key statements are supported by references __________________________________________2

5) Scientific reasoning (e.g., incorporation of appropriate evidence, such as RCTs in clinical medicine)

The article’s point s not based on appropriate arguments ________________________________0

2

Appropriate evidence is introduced selectively ________________________________________1

Appropriate evidence is generally present ___________________________________________ 2

6) Appropriate presentation of data (e.g., absolute vs relative risk; effect sizes without confidence intervals)

Data are presented inadequately ___________________________________________________0

2

Data are often not presented in the most appropriate way_______________________________1

Relevant outcome data are generally presented appropriately ___________________________

12/12

Summary
